# MicroRNAs as Diagnostic Biomarkers for Deep Vein Thrombosis

**DOI:** 10.3390/diagnostics16203351

**Published:** 2026-10-15

**Authors:** Vårin Eiriksdatter Wikan, Øyvind Øverli, Thor Ueland, Sigrid Kufaas Brækkan, Gholamreza Jafari Yeganeh, John-Bjarne Hansen, Ellen Elisabeth Brodin

**Affiliations:** 1 Thrombosis Research Group (TREC), Department of Clinical Medicine, UiT The Arctic University of Norway, N-9037 Tromsø, Norway; 2 Thrombosis Research Center (TREC), Division of Internal Medicine, University Hospital of North Norway, N-9019 Tromsø, Norway; 3 Hematological Research Group, Division of Medicine, Akershus University Hospital, N-1478 Lørenskog, Norway; 4 Research Institute of Internal Medicine, Oslo University Hospital Rikshospitalet, N-0424 Oslo, Norway; 5 Institute of Clinical Medicine, Faculty of Medicine, University of Oslo, N-0318 Oslo, Norway; 6 Department of Emergency Medicine, Akershus University Hospital, N-1478 Lørenskog, Norway

**Keywords:** microRNA, diagnostic biomarkers, deep vein thrombosis, cross-sectional study, venous thromboembolism

## Abstract

**Background/Objectives:** In patients with suspected acute deep vein thrombosis (DVT), new blood biomarkers with high specificity for DVT could potentially lower the proportion of patients requiring imaging. The plasma microRNAs (miRs) miR-134-5p, miR-103a-3p and miR-210-3p have been proposed as diagnostic biomarkers of venous thromboembolism, but their diagnostic accuracy for acute DVT has not been investigated. We aimed to assess the diagnostic performance of miR-134-5p, miR-103a-3p and miR-210-3p for DVT in patients with suspected first-lifetime acute lower-extremity DVT. **Methods:** This prospective cross-sectional study consecutively included outpatients with suspected first-lifetime acute lower-extremity DVT. Plasma levels of miRs were measured using reverse transcription quantitative polymerase chain reaction. DVT status was determined by whole-leg duplex compression ultrasonography. Diagnostic test performance was assessed by the area under the curves (AUCs) from receiver operating characteristic curves with corresponding 95% confidence intervals (95% CI). The diagnostic accuracy of D-dimer was also assessed for comparison. **Results:** Among the 360 patients included in this study, 28% had a first-lifetime acute lower-extremity DVT. The AUCs for miR-134-5p, miR-103a-3p, and miR-210-3p ranged from 0.53 to 0.57, with 95% CIs overlapping 0.50. D-dimer had a sensitivity of 97% and specificity of 41% using the cut-off value of 0.5 µg/mL. **Conclusion:** Plasma miR-134-5p, miR-103a-3p and miR-210-3p showed poor diagnostic test performance for DVT. Our findings suggest that these miRs would not be helpful in discriminating between patients in whom DVT can be safely excluded and those who require imaging.

## 1. Introduction

Diagnostic guidelines for venous thromboembolism (VTE), encompassing deep vein thrombosis (DVT) and pulmonary embolism (PE), recommend a rule-out strategy for patients with suspected VTE to ensure adequate diagnostic accuracy and cost-effectiveness [1,2,3,4]. In patients with suspected DVT, the strategy recommends that patients who are unlikely to have DVT based on a pre-test probability (PTP) score and a negative D-dimer should be ruled out from further radiological imaging [1,2]. Adherence to this strategy is shown to reduce the number of radiological procedures for DVT suspicion by approximately 30–40% [1,2]. However, physicians often deviate from this strategy [5,6], contributing to overuse of imaging procedures, increased healthcare costs, and detection of findings with uncertain clinical significance [1,7,8].

Alternative approaches aiming to optimize the rule-out strategy have been developed over the last decade, of which several appear to be superior to the currently recommended strategy [9,10]. Most alternative strategies have focused on changing the PTP score and/or D-dimer testing, either by reducing the number of items in PTP scores or utilizing adjusted D-dimer cut-off values to age or PTP results [9,10,11,12,13]. Implementation of these alternative strategies may be threatened due to mistrust of PTP and D-dimer testing among medical doctors [6,14,15]. Lack of trust in the ability of PTP and D-dimer to safely rule out VTE was an important barrier for implementation of these tests into daily practice among emergency physicians [6]. Other approaches are therefore warranted to distinguish patients with suspected DVT in whom DVT can be safely excluded without imaging from those who require imaging. Discovery of new diagnostic biomarkers with high specificity for DVT and their implementation in the current rule-out strategy for DVT suspicion would be an alternative approach [16].

MicroRNAs (miRs) are small non-coding, gene-regulating molecules that have emerged as a promising group of disease biomarkers due to their stability in plasma [17,18,19,20,21]. Previous studies have reported altered plasma levels of miR-134-5p, miR-103a-3p and miR-210-3p in patients with acute VTE compared to non-VTE controls [22,23,24,25,26,27]. Furthermore, because miR-210-3p is upregulated in venous endothelial cells under hypoxic conditions [28,29,30], we hypothesized that hypoxia distal to a DVT could lead to elevated plasma miR-210-3p levels in the systemic circulation. Although these miRs may have the potential to be diagnostic biomarkers of DVT, their diagnostic performance in patients with signs and symptoms of DVT has not yet been investigated [22,23,24,25,26,27]. Therefore, we aimed to assess the diagnostic performance of miR-134-5p, miR-103a-3p and miR-210-3p for DVT in a prospective cross-sectional study of patients with suspected first-lifetime acute lower-extremity DVT.

## 2. Material and Methods

### 2.1. Study Design and Setting

We conducted a diagnostic cross-sectional study with prospective, consecutive inclusion of outpatients with suspicion of acute lower-extremity DVT referred to the emergency department or VTE outpatient clinic at Akershus University Hospital (Norway) from 18 June 2021 to 26 June 2023. No restrictions regarding pre-test probability or D-dimer plasma levels were applied. All potentially eligible participants received oral and written study information before deciding whether they wanted to participate or not. The study was approved by the regional committee for health and research ethics (Project number: 200878) and complied with the Declaration of Helsinki and STARD (Standards for Reporting of Diagnostic Accuracy Studies) 2015 reporting items.

### 2.2. Eligibility Criteria and Study Flow

Eligible patients were defined as patients with symptoms and signs of lower-extremity DVT (such as leg pain and/or swelling), having Akershus University Hospital as their local hospital and who consented to participate before their disease status was determined. The study entry point was referral to compression ultrasonography (CUS) by primary care physicians. Patients were excluded if they were <18 or ≥80 years of age, pregnant, had an anticipated life expectancy less than 2 years or were on long-term anticoagulant treatment at enrollment. Additionally, patients with recurrent VTE, patients diagnosed with PE without concomitant DVT, and patients in need of emergency reperfusion therapy were excluded. Emergency reperfusion therapy was defined as thrombolytics (systemic or catheter-directed) or thrombectomy (mechanical or catheter-directed). We also excluded patients in whom blood sampling or CUS was not performed, patients whose miR samples did not pass the assay quality control and patients who had CUS performed or miR samples drawn > 24 h after hospital arrival.

Single point-of-care whole-leg CUS with color Doppler (duplex CUS) performed by emergency physicians was used as the reference standard in this study. Patients with inconclusive CUS results were referred to the radiological department at Akershus University Hospital for a second and definitive examination. CUS was the only accepted reference standard, as multiple reference standards would increase the risk of bias [31,32]. CUS has a sensitivity of 94% and specificity of 97% for lower-extremity DVT and was chosen over computed tomography (CT) venography as it is the most commonly used and avoids exposure to radiation and contrast injections [1,2,33,34]. Patients with CUS-verified proximal lower-extremity DVT (uncompressible popliteal vein or more proximal deep veins) or distal lower-extremity DVT (uncompressible deep veins distal to the popliteal vein) with or without concomitant PE in need of at least three months of anticoagulant treatment were regarded as patients with DVT (disease of interest) in this study, whereas all other patients were regarded as non-DVT patients. The medical records of all non-DVT patients were reviewed three months after study enrollment to search for missed VTEs.

The three plasma miRs, miR-134-5p, -103a-3p and -210-3p, were regarded as study index tests. Plasma D-dimer was measured as part of routine clinical blood work, and its diagnostic performance was assessed as a clinical benchmark. Both blood sampling and CUS were performed shortly after hospital admission and always within 24 h. D-dimer levels were measured by the laboratory staff at the Department of Medical Biochemistry at Akershus University Hospital immediately after blood sampling, while the miRs samples were biobanked and shipped to QIAGEN Genomic Services (Hilden, Germany) for analyses upon inclusion of all patients. The personnel measuring plasma miR levels and performing CUS were blinded to one another’s results. However, CUS operators had access to the D-dimer results. Other therapeutic or diagnostic interventions of eligible patients were not controlled in this study, including the administration of a single dose of anticoagulant medication prior to blood sampling and CUS. In Norway, the use of single-dose anticoagulation when VTE is suspected is common practice if the waiting time for radiological procedures is anticipated to be several hours.

### 2.3. Sampling and Measurement of microRNAs and D-Dimer

Non-fasting blood samples were drawn from an antecubital vein using a 21-gauge needle into citrate tubes shortly after hospital arrival, either together with routine blood work or as an additional sampling. Blood samples were centrifuged at 2500× *g* for 15 min at room temperature. Platelet-poor plasma was aliquoted and then stored at −80 °C. Plasma levels of miR-134-5p, miR-103a-3p and miR-210-3p were measured using quantitative polymerase chain reaction (PCR). Frozen plasma samples were shipped on dry ice to QIAGEN Genomic Services (Hilden, Germany) for measurement of the miRs. Upon arrival, plasma samples were thawed on ice and centrifuged at 3000× *g* for 5 min at 4 °C using a microcentrifuge. Samples were prepared and RNA was extracted using miRNeasy Serum/Plasma Advanced Kit v.1 (QIAGEN, Hilden, Germany) [35]. The purified total RNA was eluted in a final volume of 50 µL of which 2 µL was reverse transcribed into complementary DNA in 10 µL reactions using miRCURY LNA RT Kit (QIAGEN, Hilden, Germany). Complementary DNA was then diluted 50 times and assayed in 10 µL reactions as described in the miRCURY LNA miR PCR protocol [36]. Each miR, including index test-miRs and miRs for normalization and quality control, was then assayed once for quantitative real-time PCR on the miRNA Ready-to-Use PCR custom panel miRCURY LNA SYBR Green master mix (QIAGEN, Hilden, Germany) [36]. Negative controls without templates from the reverse transcription reaction were performed and profiled like the other samples. The amplification was performed in a LightCycler^®^ 480 Real-Time PCR System (Roche Diagnostics, Basel, Switzerland). Of note, the raw quantification cycle (Cq) values of the four index test miRs were normalized by the expression of miR-425-5p using the 2ΔCt-method, as this miR has been previously used as a reference in several miR studies and is stably expressed in plasma [37,38,39]. The manufacturer’s data quality control was assessed by melting curve analyses, spike-in miRs for RNA isolation and complementary DNA synthesis that indicated outliers, negative controls, and hemolysis, which was defined as the delta Cq (Cq miR-23a–Cq miR-451) > 7.0 [40].

D-dimer was analyzed immediately after blood sampling at the Department of Multidisciplinary Laboratory Medicine and Medical Biochemistry at Akershus University Hospital using the Tina-quant^®^ D-dimer Gen. 2 assay on Cobas t711 (D-DI2; Roche Diagnostics, Basel, Switzerland). A positive D-dimer test result for VTE using this assay was defined as ≥0.5 µg/mL FEU (Fibrinogen Equivalent Units).

### 2.4. Statistical Analyses

Sample size was calculated based on the minimum effect size of miR-145-5p that was considered clinically significant, as described in a previous diagnostic accuracy study [39]. Clinical significance was defined as the smallest effect size, Cohen’s d of 0.57, able to reduce the main proportion of CUS referrals due to DVT suspicion by 10%. The anticipated proportion of required CUS was based on four previous diagnostic DVT studies [12,41,42,43]. Using this method, the required sample size was 94 DVT patients and 235 non-DVT patients.

To evaluate group differences in plasma miR levels between DVT and non-DVT patients, we used Wilcoxon rank-sum tests with a significant *p*-value threshold of *p* < 0.05 and the effect size measure Cohen’s d with corresponding 95% confidence intervals (95% CI) estimated by bootstrapping of 5000 permutations. Typically, a Cohen’s d effect size of 0.2 or lower would be considered small and an effect size of 0.8 or higher would be considered large [44]. To assess the diagnostic accuracy of the miRs, logistic receiver operating characteristic (ROC) curves with corresponding 95% CIs were generated and their area under the curves (AUCs) were calculated to assess the test performance of miRs for DVT [45]. A ROC curve was also generated for D-dimer, and the calculation of sensitivity, specificity and predictive values for D-dimer was performed using the cut-off value of 0.5 µg/mL. Sex differences in plasma levels of another VTE-related miR (miR-145-5p) have previously been reported [39], and whether anticoagulants alter the plasma levels of miR-134-5p, miR-103a-3p or miR-210-3p is unknown. We therefore performed sensitivity analyses stratified by sex and by whether a single dose of anticoagulation medication was administered or not. To ease diagnostic interpretation, the ROC curves of the miRs were flipped using the negative normalized miR values if the plasma levels of miR were lower in the DVT group compared to the non-DVT group. All statistical analyses were performed using Stata version 18.0 (Stata Corporation LP, College Station, TX, USA). Illustrations of the distribution of plasma values, median plasma levels and interquartile range (IQR) of miRs and D-dimer were made by GraphPad Prism version 6.05 for Windows, GraphPad Software, Boston, MA, USA. A detailed description of all data extraction elements (clinical characteristics, etc.) can be found in a previous publication [46].

## 3. Results

**Figure 1:**
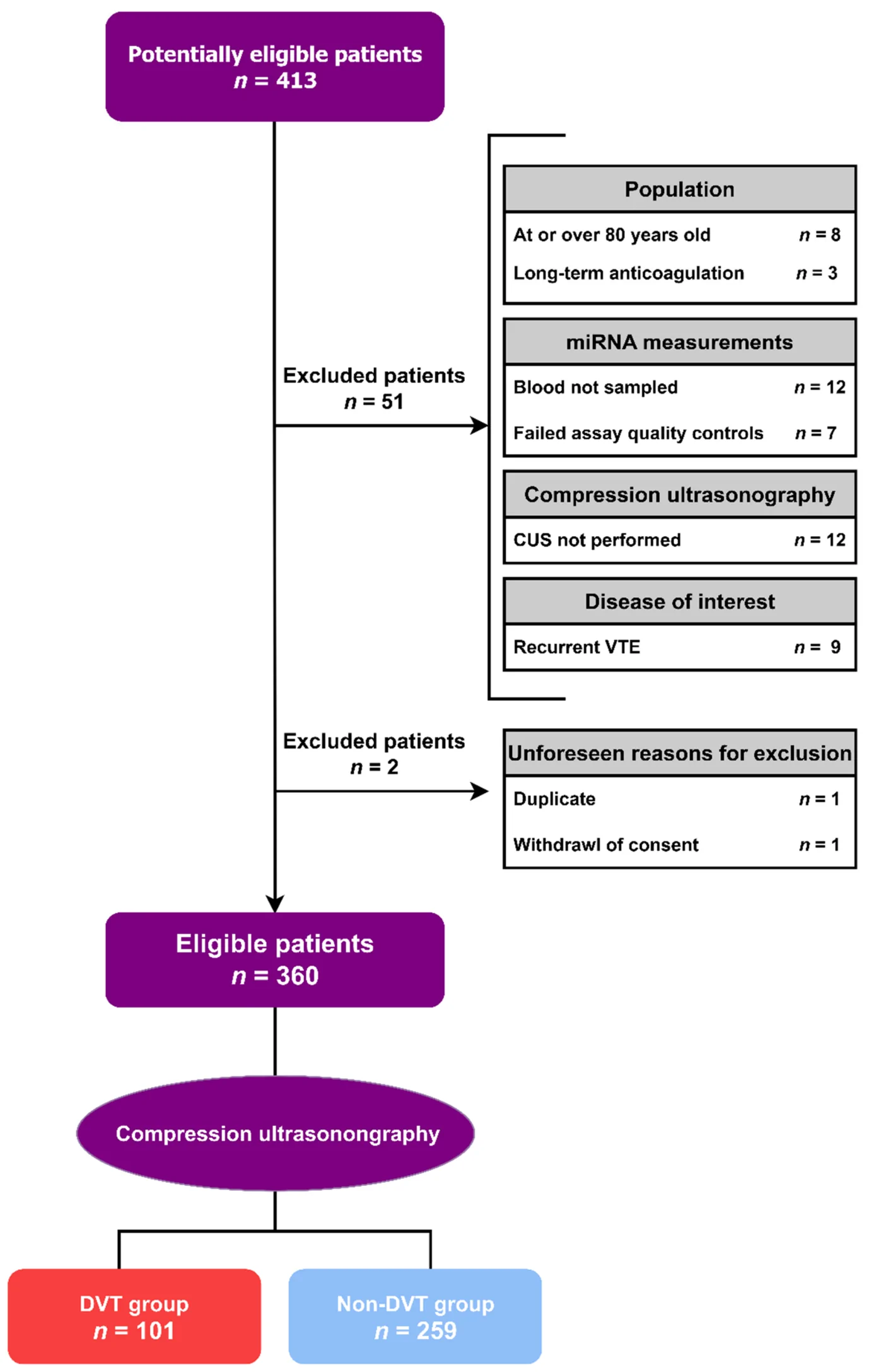
Flow of enrolled patients. Abbreviations: deep vein thrombosis (DVT), compression ultrasonography (CUS) and venous thromboembolism (VTE).

The flow of participants and the reasons for study exclusion are summarized in Figure 1. Among the 413 potentially eligible participants, 53 were excluded (Figure 1). Thus, 360 patients were included in the analyses, of whom 101 patients (28.1%) had DVT.

The baseline and clinical characteristics of the participants are shown in Table 1. Of note, the proportion of males was larger in the DVT group (63%) than in the non-DVT group (40%), and the median age was 58 years in both groups. The frequency of VTE risk factors and co-morbidities was low and similar between groups, except for surgery/trauma, family history of VTE and overweight, which were more prevalent among DVT patients (Table 1). Administration of a single dose of anticoagulation prior to the diagnostic work-up was more prevalent in the DVT group (38%) than in the non-DVT group (24%). A similar proportion of patients (31% in the DVT group and 36% in the non-DVT group) had a PTP score (the full or simplified version of the Wells score for DVT) reported in their medical record. Regarding the spectrum of DVT severity, 73% had a proximal DVT and 24% of all DVT patients had a concomitant PE confirmed by computed tomography (CT) pulmonary angiography. The non-DVT group was coded with 46 different ICD-10 diagnoses (Supplementary Table S1). The three most common differential diagnoses were “pain in limb” (54%), “rupture of popliteal/Baker’s cyst” (12%) and “superficial vein thrombosis” (8.5%). Review of medical records of non-DVT patients three months after study inclusion revealed the occurrence of three additional DVTs and one additional PE. These four patients with missed VTEs at enrollment remained non-DVT patients in our statistical analyses.

**Figure 2:**
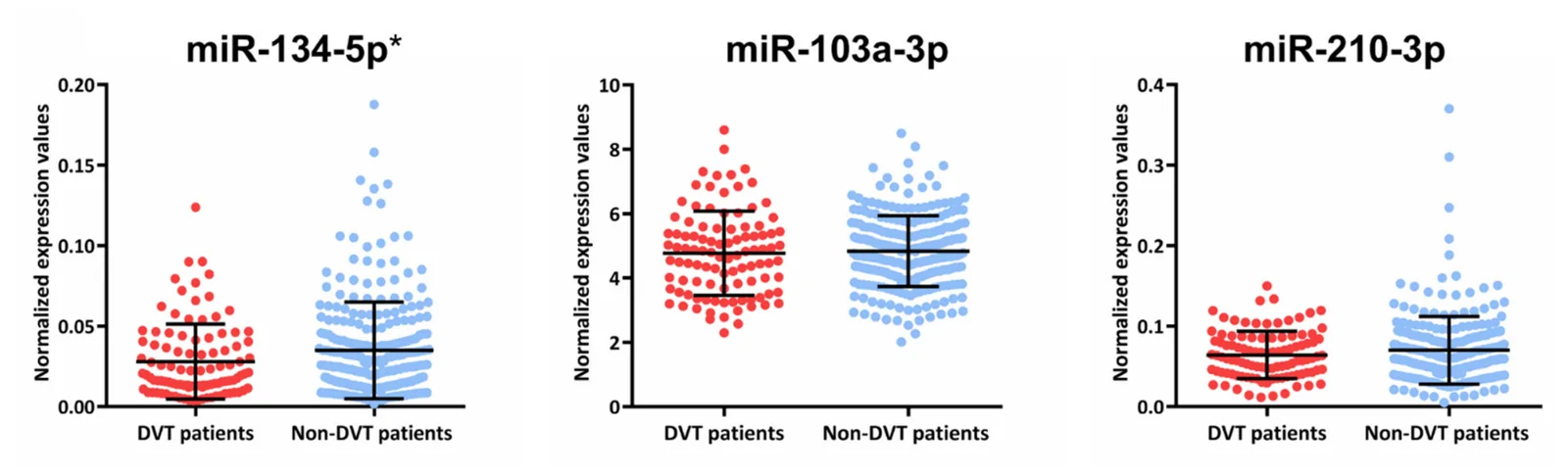
Normalized plasma miR levels in DVT and non-DVT patients. This panel illustrates the distribution with corresponding medians and interquartile ranges of plasma concentrations of the miRs in deep vein thrombosis (DVT) patients and non-DVT patients. The symbol “*” indicates significant differential expression between the two groups (*p* < 0.05).

**Figure 3:**
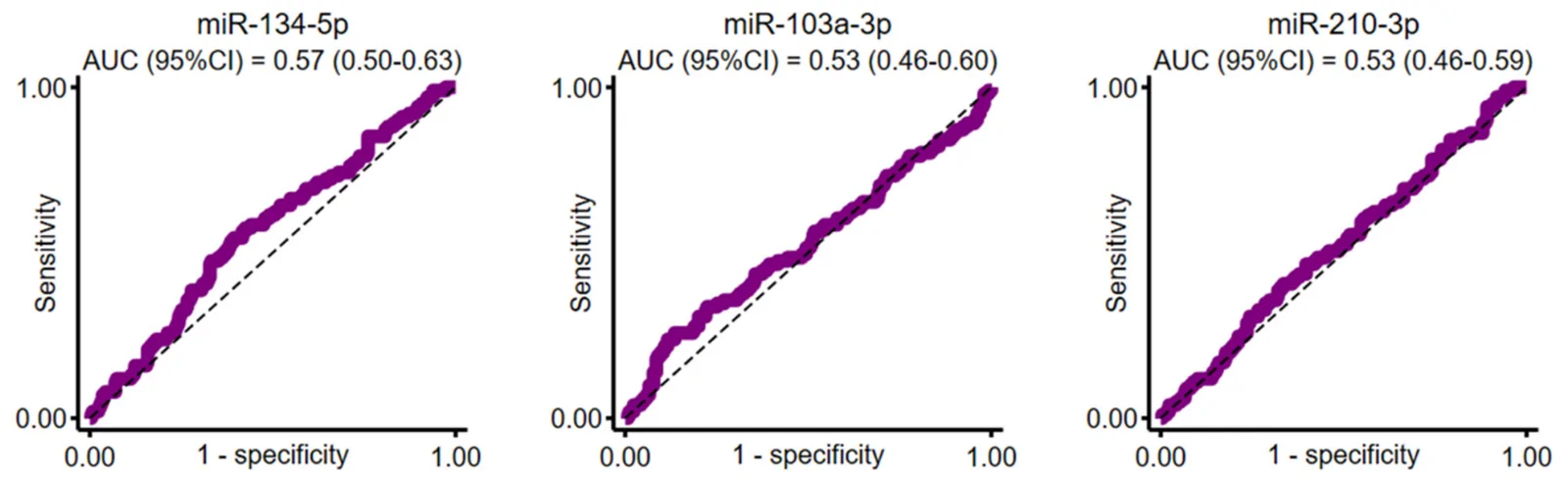
Diagnostic performance of microRNAs. The figure illustrates the area under curve (AUC) of receiver operating characteristic curves with corresponding 95% confidence intervals (95% CI) of the microRNAs (miRs) miR-134-5p, miR-103a-3p and miR-210-3p.

The median normalized plasma levels of miR-134-5p, miR-103a-3p and miR-210-3p were slightly lower in the DVT group compared to the non-DVT group, of which only miR-134-5p had a significant group difference (*p*-value = 0.047) (Figure 2). The sparse group differences were also demonstrated by Cohen’s d for the miRs of −0.25 (95% CI −0.44 to −0.05) for miR-134-5p, −0.06 (95% CI −0.31 to 0.19) for miR-103a-3p and −0.15 (95% CI −0.35 to 0.04) for miR-210-3p. The AUCs of the miR ROCs were 0.57 (95% CI 0.50 to 0.63) for miR-134-5p, 0.53 (95% CI 0.46 to 0.60) for miR-103a-3p and 0.53 (95% CI 0.46 to 0.59) for miR-210-3p (Figure 3). D-dimer was measured in all DVT patients (*n* = 101) and in 98% of non-DVT patients (*n* = 255) and yielded a Cohen’s d of 1.66 (95% CI 1.94 to 1.34) and an AUC of 0.92 (95% CI 0.89 to 0.95). With the cut-off value of 0.5 µg/mL, D-dimer showed a sensitivity of 97%, specificity of 41%, negative predictive value of 97% and positive predictive value of 39% for DVT (Supplementary Figure S1). Our sensitivity analyses found similar Cohen’s d and AUC values across sexes, as well as in those who received a single dose of anticoagulation medication and those who did not (Supplementary Table S2).

## 4. Discussion

In this study, we investigated the diagnostic accuracy of three miRs in plasma (miR-134-5p, miR-103a-3p and miR-210-3p) for first-lifetime acute lower-extremity DVT in consecutively enrolled outpatients with signs and symptoms of DVT. In total, 28% had a DVT confirmed by whole-leg duplex CUS. The group differences in plasma miRs were minimal between DVT and non-DVT patients. Accordingly, the miRs were unable to discriminate between DVT and non-DVT conditions, as indicated by AUCs ranging from 0.53 to 0.57 and 95% CI intervals overlapping 0.50. The range of different DVT severities and differential diagnoses among eligible patients appeared representative of clinical practice, as supported by the test performance of D-dimer with a negative predictive value of 97% and a positive predictive value of 39% in our patient sample.

Previous studies reported distinct differences in plasma levels of miR-134-5p [23,24,25,26,27], miR-103a-3p [22] and miR-210-3p [23] between patients with acute VTE and controls, along with promising test performance of miR-134-5p for acute PE. In a study by Peng et al., the diagnostic accuracy of serum miR-134-5p for acute PE was assessed in prospectively recruited inpatients with acute exacerbation of chronic obstructive pulmonary disease (COPD) referred to CT pulmonary angiography for PE suspicion [25]. Only 52 out of 217 enrolled COPD patients were included in the analyses, as most patients were excluded due to co-morbidities (162 patients) [25]. Among the included patients, 13 were diagnosed with PE (39 non-PE patients). Plasma miR-134-5p levels were significantly lower in the PE group compared to the non-PE group with a correspondingly high diagnostic accuracy for acute PE (AUC of 0.93) [25]. Similarly, a case-control study showed lower plasma miR-103a-3p levels in 90 patients with acute lower-extremity DVT compared to 20 healthy controls [22]. Regarding miR-210-3p, lowered plasma levels, though not significant, were found for acute PE in a small case-control study by Zhou et al., including 37 PE cases and 37 healthy controls [23]. In the same case-control study, a decreased level of plasma miR-134-5p was found in PE [23]. In contrast, smaller case-control studies reported higher levels of miR-134-5p in plasma or serum in patients with acute PE compared to healthy controls with moderate-to-high diagnostic accuracy for PE (AUCs ranging from 0.83 to 0.90) [24,26,27].

No previous studies have, to the best of our knowledge, investigated the test performance of these miRs for acute DVT. We found that none of the plasma miRs (miR-134-5p, miR-103a-3p or miR-210-3p) displayed clinically useful test performance for patients with suspected acute DVT. For miR-210-3p, the findings contradict our hypothesis that elevation of plasma miR-210-3p levels in the systemic circulation, caused by hypoxia distal to the venous occlusion, would be specific enough to discriminate between DVT and non-DVT patients with similar signs and symptoms. The lack of discriminatory power for miR-134-5p and miR-103-3p as diagnostic biomarkers for acute lower-extremity DVT is in contrast with previous promising findings [22,23,24,25,26,27].

The apparent discrepancies between our findings and previous studies might be explained by study design (cross-sectional vs. case-control), sample size, pre-analytical handling of blood samples, and the fact that PE was the VTE presentation of interest for miR-134-5p rather than DVT [32,45,47]. The study by Peng et al. [25] utilized serum, where ex vivo platelet degranulation could artificially augment circulating miR-134-5p levels, and this artifact may be amplified in thrombosis-related conditions due to hyperactive platelets. Our two-step centrifugation protocol isolated platelet-poor plasma, thereby avoiding clotting-induced cellular release and mitigating potential artifacts associated with lower-speed plasma processing. Furthermore, the diagnostic-test accuracy is shown to be substantially inflated in diagnostic case-control studies, especially those using healthy participants as controls, compared with studies recruiting symptomatic patients suspected of having the disease of interest from the same source population [47]. The inconsistent direction of change in plasma miR-134-5p levels in acute PE, the small sample sizes, and the omission of participants with differential diagnoses of PE in previous studies, suggests that their discrepancy with our findings could be attributed to restricted disease spectrums in these studies, driven by their study design, rather than a higher diagnostic accuracy for PE than DVT for the investigated miRs [32,45,47].

This study has some limitations. The DVT prevalence of 28% in this study was higher than what has been observed in areas with low compliance with the diagnostic VTE rule-out strategy [8,34,48]. Since CUS referral was the entry point of the study, patients with low PTP and low D-dimer could potentially have been missed if they were not referred to CUS [1]. Such missingness could lead to a higher DVT prevalence, as well as the underrepresentation of patients with less severe differential diagnoses of DVT, potentially deflating the diagnostic accuracy of miRs for DVT [45]. However, only 34% of the physicians at Akershus University Hospital reported a PTP score in the CUS referrals, implying low compliance with the rule-out strategy. Furthermore, the spectrum of differential diagnoses and the D-dimer test performance in our study were similar to those of other studies with consecutive sampling of patients with suspected DVT [12,34,49,50,51]. This indicates that the use of CUS referrals as the study entry point was likely not a major contributor to our higher-than-expected DVT prevalence. Alternatively, the high DVT prevalence could be explained by patients’ motivation for participation [52]. Patients experiencing more severe DVT symptoms (i.e., having higher PTP for DVT) might be more motivated for study participation and thereby be more likely to consent compared to patients with less severe DVT symptoms (i.e., having lower PTP for DVT) [52]. Whole-leg CUS has reported variable sensitivity for lower-extremity DVT [7], which might have led to some degree of misclassification of the outcome of interest. However, the three-month failure rate of whole-leg CUS for DVT was low (1.5%), indicating that the impact of this misclassification was limited [7]. Furthermore, the time course of DVT may influence plasma miR levels, and the diagnostic performance of the investigated miRs may differ depending on the time from symptom onset to blood sampling. However, as the time-course from onset of symptoms to seeking medical attention for a DVT may vary between individuals (patient delay), the ideal biomarker for a DVT should not be dependent on the stage of DVT development. Finally, the exclusion of specific VTE subgroups, such as atypical DVTs and recurrent VTEs, as well as the exclusion of pregnant patients, patients with life expectancy of less than 2 years and patients younger than 18 or older than 80 years of age, may have limited the generalizability of our results to these subgroups.

In conclusion, the overall poor test performance of miR-134-5p, miR-103a-3p, and miR-210-3p for DVT in our study indicates that these miRs are not useful diagnostic blood biomarkers for first-lifetime acute lower-extremity DVT.

## Figures and Tables

**Table 1:** Demographic and clinical characteristics of eligible patients. Abbreviations DVT: deep vein thrombosis, COPD: chronic obstructive pulmonary disease, IQR: interquartile range, PE: pulmonary embolism and VTE: venous thromboembolism. ^a^ Hormonal therapy was defined as estrogen-containing oral contraceptives, hormonal replacement therapy with estrogens or selective estrogen receptor modulators. ^b^ Overweight was defined as BMI at or above 25. ^c^ Patients whose pregnancies were unrecognized at study enrollment, but confirmed shortly after, and recorded during medical record review. ^d^ Single dose of either low-molecular-weight heparin or direct oral anticoagulant.

	DVT Patients (*n* = 101)	Non-DVT Patients (*n* = 259)
Age (years), median (IQR)	58 (48–67)	58 (49–70)
Male sex, *n* (%)	64 (63%)	104 (40%)
Symptom duration > 4 days, *n* (%)	60 (59%)	152 (59%)
Surgery or trauma, *n* (%)	17 (17%)	28 (11%)
Family history of VTE, *n* (%)	18 (18%)	18 (7%)
Hormonal therapy ^a^, *n* (%)	7 (7%)	12 (5%)
Overweight ^b^, *n* (%)	38 (38%)	68 (26%)
Pregnancy ^c^ or postpartum, *n* (%)	1 (1%)	4 (2%)
Active cancer, *n* (%)	8 (8%)	23 (9%)
Previous myocardial infarction, *n* (%)	5 (5%)	16 (6%)
Previous stroke, *n* (%)	2 (2%)	5 (2%)
Heart failure, *n* (%)	1 (1%)	3 (1%)
COPD, *n* (%)	6 (6%)	11 (4%)
Single dose of anticoagulation ^d^, *n* (%)	38 (38%)	61 (24%)
Reported Wells score, *n* (%)	32 (31%)	92 (36%)
D-dimer measured, *n* (%)	101 (100%)	255 (98%)
Isolated DVT, *n* (%)	77 (76%)	N/A
DVT and PE, *n* (%)	24 (24%)	N/A
Proximal DVT, *n* (%)	74 (73%)	N/A
Distal DVT, *n* (%)	27 (27%)	N/A
Missed DVT, *n* (%)	N/A	3 (1.2%)
Missed PE, *n* (%)	N/A	1 (0.4%)

## Data Availability

Anonymized datasets used and/or analyzed during the current study are available from the corresponding author on reasonable request.

## Supplementary Materials

The following supporting information can be downloaded at: https://www.mdpi.com/article/10.3390/diagnostics16203351/s1, Supplementary Table S1: The diagnoses of eligible patients with excluded DVT diagnoses by compression ultrasonography sorted alphabetically; Supplementary Table S2: Cohen’s d and area under the curve (AUC) values from receiver operating characteristic (ROC) curves of miR-134-5p, miR-103a-3p and miR-210-3p stratified by sex and whether a single dose of anticoagulation medication was administrated; Supplementary Figure S1: Plasma levels and diagnostic performance of high-sensitive D-dimer.
