# Supplementary material for: MicroRNAs as Diagnostic Biomarkers for Deep Vein Thrombosis
Source: Diagnostics (Basel). 2026 Oct 15;16(20):3351. doi: 10.3390/diagnostics16203351 (PMC13703451; doi:10.3390/diagnostics16203351)
Supplement: Supplementary file 1 [file diagnostics-16-03351-s001.zip › diagnostics-4593534-supplementary.pdf]

Supplementary material

Supplementary Table S1: *The diagnoses of eligible patients with excluded DVT diagnoses by compression ultrasonography sorted alphabetically. Abbreviations DVT: deep vein thrombosis, ICD: international classification of disease and PE: pulmonary.*

ICD-10 diagnoses of non-DVT-patients (*n*=259)		
M76.6	Achilles tendinitis	3
I21.4	Acute subendocardial myocardial infarction[1]	1
L50.0	Allergic urticaria	1
I72.4	Aneurysm and dissection of artery of lower extremity	1
M13.96	Arthritis, unspecified;calf/knee	1
D17.2	Benign lipomatous neoplasm of skin and subcutaneous tissue of limbs	1
M77.3	Calcaneal spur	2
L03.9	Cellulitis, unspecified	3
S80.0	Contusion of knee	1
S80.1	Contusion of other and unspecified parts of lower leg	1
R06.0	Dyspnoea[1]	1
A46	Erysipelas	2
M10.0	Idiopathic gout	6
M10.07	Idiopathic gout;ankle/foot/toe	1
M12.46	Intermittent hydrarthrosis;calf/knee	1
C77.2	Intra-abdominal lymph nodes	1
R60.0	Localized oedema	5
R22.4	Localized swelling, mass and lump, lower limb	4
R22.9	Localized swelling, mass and lump, unspecified	1
A69.2	Lyme disease	1
I89.0	Lymphoedema, not elsewhere classified	1
M62.6	Muscle strain	1
M79.16	Myalgia;calf/knee	2
R60.9	Oedema, unspecified	1
M23.2	Other meniscus derangements	1
M12.8	Other specific arthropathies, not elsewhere classified	1
D69.8	Other specified haemorrhagic conditions	1
M25.5	Pain in joint	2
M25.56	Pain in joint;calf/knee	2
M79.67	Pain in limb[2]	8
M25.57	Pain in limb;ankle/foot/toe[2]	1
M79.66	Pain in limb;calf/knee[2]	129
M79.65	Pain in limb;pelvis/thigh[2]	1
M76.7	Peroneal tendinitis	1

I80.2	Phlebitis and thrombophlebitis of other deep vessels of lower extremities[3]	8
I80.8	Phlebitis and thrombophlebitis of other sites[3]	7
I80.0	Phlebitis and thrombophlebitis of superficial vessels of lower extremities[3]	6
I80.9	Phlebitis and thrombophlebitis of unspecified site[3]	1
M79.55	Residual foreign body in soft tissue;pelvis/thigh	1
M66.0	Rupture of popliteal cyst	32
C79.5	Secondary malignant neoplasm of bone and bone marrow	1
M79.9	Soft tissue disorder, unspecified	1
R23.3	Spontaneous ecchymoses	1
M71.2	Synovial cyst of popliteal space [Baker]	4
I83.9	Varicose veins of lower extremities without ulcer or inflammation	6
I87.2	Venous insufficiency (chronic)(peripheral)	1
[1] = patient had symptoms of both DVT and PE.		
[2] = sum of diagnoses related to “pain in limb” from the pelvis to toes: 139		
[3] = sum of diagnoses related to superficial vein thrombosis: 22		

Supplementary Table S2: *Cohen’s d and area under the curve (AUC) values from receiver operating characteristic (ROC) curves of miR-134-5p, miR-103a-3p and miR-210-3p stratified by sex and whether a single dose of anticoagulation medication was administrated.*

		**Cohen’s d**	**AUCs of ROC curves**
**Sex**			
**Males**	miR-134-5p	-0.31 (-0.56 to -0.07)	0.59 (0.50 to 0.68)
	miR-103a-3p	-0.13 (-0.47 to 0.20)	0.56 (0.47 to 0.66)
	miR-210-3p	-0.18 (-0.44 to 0.09)	0.52 (0.43 to 0.61)
**Females**	miR-134-5p	-0.14 (-0.49 to 0.20)	0.53 (0.43 to 0.62)
	miR-103a-3p	0.10 (-0.49 to 0.29)	0.54 (0.43 to 0.65)
	miR-210-3p	-0.09 (-0.49 to 0.21)	0.51 (0.40 to 0.61)
**Anticoagulation**			
**Single dose of anticoagulation**	miR-134-5p	-0.10 (-0.50 to 0.28)	0.56 (0.44 to 0.68)
	miR-103a-3p	0.07 (-0.48 to 0.34)	0.52 (0.40 to 0.64)
	miR-210-3p	0.08 (-0.30 to 0.46)	0.50 (0.38 to 0.62)
**No anticoagulation**	miR-134-5p	-0.33 (-0.54 to -0.13)	0.58 (0.50 to 0.66)
	miR-103a-3p	-0.07 (-0.40 to -0.26)	0.53 (0.44 to 0.62)
	miR-210-3p	-0.17 (-0.39 to 0.05)	0.54 (0.46 to 0.62)

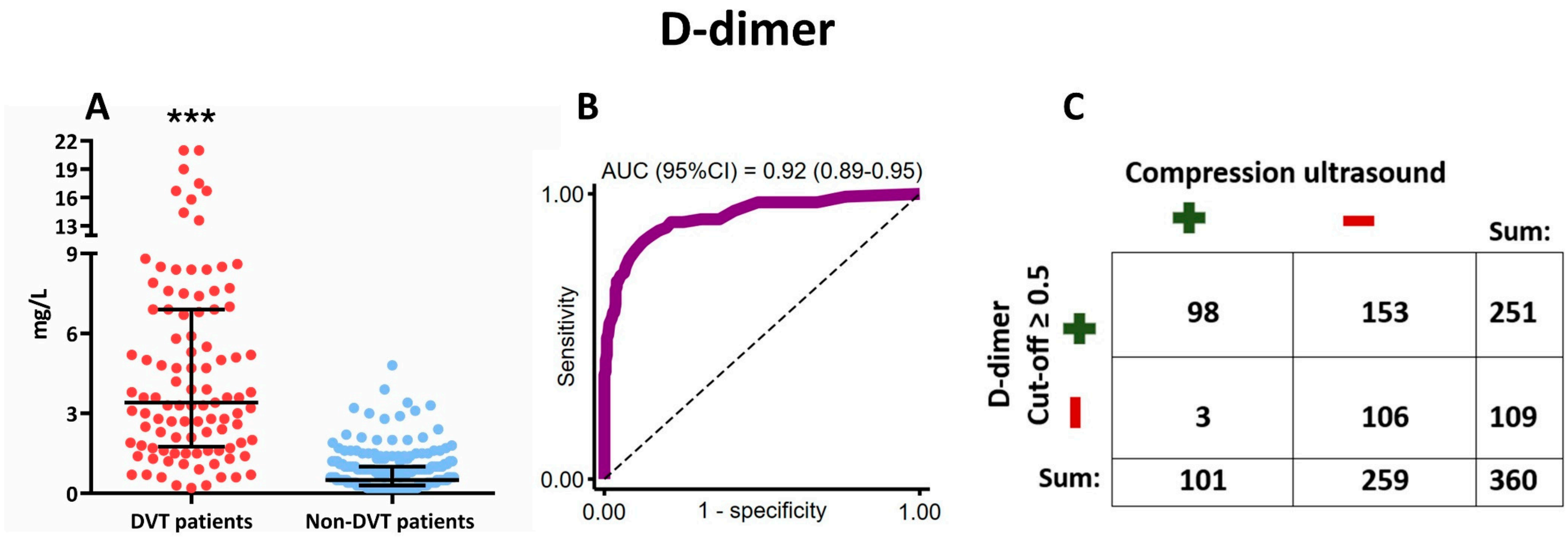

Supplementary Figure S1: *Plasma levels and diagnostic performance of high-sensitive D-dimer. A) Distribution of plasma D-dimer concentrations (µg fibrinogen equivalent units (FEU) /mL) with their corresponding median and interquartile range in deep vein thrombosis (DVT) patients and non-DVT patients. The symbol " *** " indicates a significant difference in plasma concentration between groups (p-value < 0.001). B) The receiver operating characteristics curve of D-dimer and its area under the curve (AUC) with corresponding 95% confidence interval (95%CI). C) Contingency table of D-dimer using the assay manufacturer's recommended cut-off for venous thromboembolism of 0.5 µg FEU/mL.*
